# Dynamic energy performance assessment using a digital shadow for sludge incineration in wastewater treatment plants

**DOI:** 10.1038/s41598-026-57835-1

**Published:** 2026-06-18

**Authors:** Behrouz Adibimanesh, Sylwia Polesek-Karczewska, Paulina Hercel, Atieh Khodadadi, Sanja Lazarova-Molnar

**Affiliations:** 1 Institute of Fluid Flow Machinery, Polish Academy of Sciences, Gdańsk, Poland; 2 Institute of Applied Informatics and Formal Description Methods, Karlsruhe Institute of Technology, Karlsruhe, Germany; 3 The Maersk Mc-Kinney Moller Institute, University of Southern Denmark, Odense, Denmark

**Keywords:** Digital shadow, Dynamic system, Energy performance, Simulation, Anylogic, Energy science and technology, Engineering, Environmental sciences

## Abstract

The rapid growth of the global economy and rising energy costs have underscored the need for efficient energy utilization across various sectors. To address this, we developed a novel data-informed digital shadow framework, enhancing energy management within a closed-loop control system, particularly in wastewater treatment facilities. Our approach centers on reducing biogas consumption in the fluidized bed reactor, a crucial component of the incineration process. The digital shadow simulates the entire incineration section by extracting model parameters for key components, including the furnace, dryer, mixer, and heat exchangers, from both physical models and operational data. System-level simulation was performed in AnyLogic software, integrating analytical calculations with data from positioning sensors. The model was informed by operational plant data and validated against historical system data. Evaluation of five operating scenarios demonstrated the capability of the digital shadow to capture system behavior and support operational decision-making. The evaluation of dynamic energy performance identified the most effective scenario, achieving a 40% reduction in biogas consumption while maintaining furnace operation within the temperature range specified by European standards. These findings demonstrate the effectiveness of the digital shadow approach and highlight its potential to deliver substantial annual cost savings in wastewater sludge incineration systems.

## Introduction

Population and economic growth are driving a rapid surge in global energy demand. Worldwide energy consumption in the industrial sector is projected to grow at an average annual rate of 1.4%^1^. Uncertainty over fossil fuel reserves and rising energy costs have raised energy security concerns^2^. At the same time, adopting green energy and reducing waste are vital for sustainability^3^. Consequently, improving energy efficiency is now a key strategy to cut emissions, boost security, and enhance production performance^2^. In response, the Fourth Industrial Revolution emphasizes smart systems that enable energy sustainability through the full digitization and integration of industrial value chains^4^. Energy efficiency in industrial systems is a critical aspect of modern manufacturing and production processes, aiming to reduce energy consumption while maintaining or enhancing productivity^5^. The drive towards energy-efficient industrial systems encompasses a wide range of practices, including the optimization of manufacturing processes^6^, the adoption of energy-efficient technologies^7^, and the implementation of energy management systems^8^. However, achieving higher levels of energy efficiency in industrial systems is not without its challenges. These challenges include the high initial cost of implementing energy-efficient technologies, the changes in existing systems, and a lack of understanding of the long-term benefits of energy efficiency. To effectively address these challenges, researchers recommend adopting a data-driven approach^9^.

Among the various energy-intensive processes in wastewater treatment, sludge management remains a significant contributor to overall energy consumption. One very often used method for reducing the volume and hazardous nature of sewage sludge is incineration. This process particularly popular in regions where land availability is limited, environmental regulations are strict, and energy recovery is prioritized. The primary reasons for sludge incineration include substantial volume reduction, elimination of pathogens and harmful organic compounds, and the minimization of landfill use. Incineration facilitates more efficient waste utilization and offers potential for energy recovery by further combustion process. However, this process also poses challenges, such as high operational costs, stringent emission regulations, and the need for careful handling of residual ash.

With regard to wastewater treatment systems, there are a few works suggesting solutions for lowering energy consumption. These include traditional approaches such as statistical analysis and numerical modeling, as well as advanced methodologies incorporating artificial intelligence (AI) and machine learning (ML) techniques. The work of Sun et al.^10^ gathers the technologies and methods used for energy saving in wastewater treatment plants. It highlights the challenges of the wastewater treatment industry and the benefits of energy recovery technologies, especially those based on green energy. The authors discuss various solutions. One of them is renewable sources applications, e.g. solar and wind energy, heat pumps, biomass, etc. in order to support the wastewater treatment plant (WWTP) energetic demands. Another widely discussed aspect in this article is the WWTP processes evaluation, control, and optimisation. Sludge treatment is a process of sludge stabilisation, reduction, and harmless treatment. Next, the disposal includes sludge landfill, land use, composting, and thermochemical treatment technologies. This paper summaries the methods for energy consumption reduction at almost every step of the whole wastewater utilisation process. Another work of Di Fraia et al.^11^ developed a method to define energy performance indicators, related to the overall energy consumption, and then couple the indicators with removal efficiency. The analysis is based on the assumption that in order to improve pollutants removal, additional, more sophisticated processes are needed, and therefore energy consumption might increase. Therefore, the authors decided to analyse not only energy consumption but its consumption in relation to the efficiency of the WWTP in terms of water cleaning. A large database of data from about 300 wastewater treatment plants, gathered from various sources, was used for the analysis. The authors tested their models with the existing wastewater plant in Italy and demonstrated that their approach can be useful for assessing potential energy-saving solutions.

The work of Al-Dahidi et al.^12^ presents a comprehensive approach for assessing the feasibility of waste heat recovery (WHR) in wastewater treatment plants (WWTPs) with two main objectives. First, an artificial neural network (ANN) model is developed to predict WHR potential based on operational data. Second, an economic analysis is conducted, focusing on the integration of organic rankine cycle (ORC) and seasonal thermal energy storage systems. While the study highlights the benefits of WHR systems in WWTPs, including energy efficiency, cost savings, and environmental improvements, it does not address the integration of advanced control strategies for optimizing ORC performance under varying operating conditions. Yu et al.^13^ use statistical regression methods for analyzing the possibilities of controlling and reducing energy consumption of municipal wastewater treatment plants. The authors based on a dataset of daily records gathered within one year, including the energy consumption of unit chemical oxygen demand, which was a measure of energy consumption for pollutants’ removal. Using Bayesian semi-parametic quantile regression, the authors concluded that the most influential parameters in terms of energy consumption are chromium concentration and wastewater temperature. Ding et al.^14^ presented a controlled object model for municipal solid waste incineration (MSWI). The model details the MSWI process flow, dividing the incinerator into distinct regions and constructing material and energy balance equations for each subsystem. It consists of three main parts: the analysis of the MSWI process based on a grate incinerator, the division of solid and gas phases to develop material balance equations, and the deduction of energy transfer processes using thermodynamic principles. The model was validated using data from an MSWI plant in Beijing, China. Although the model provides a solid foundation for MSWI process control, it does not address the system’s optimal performance or operating point.

**Fig. 1:**
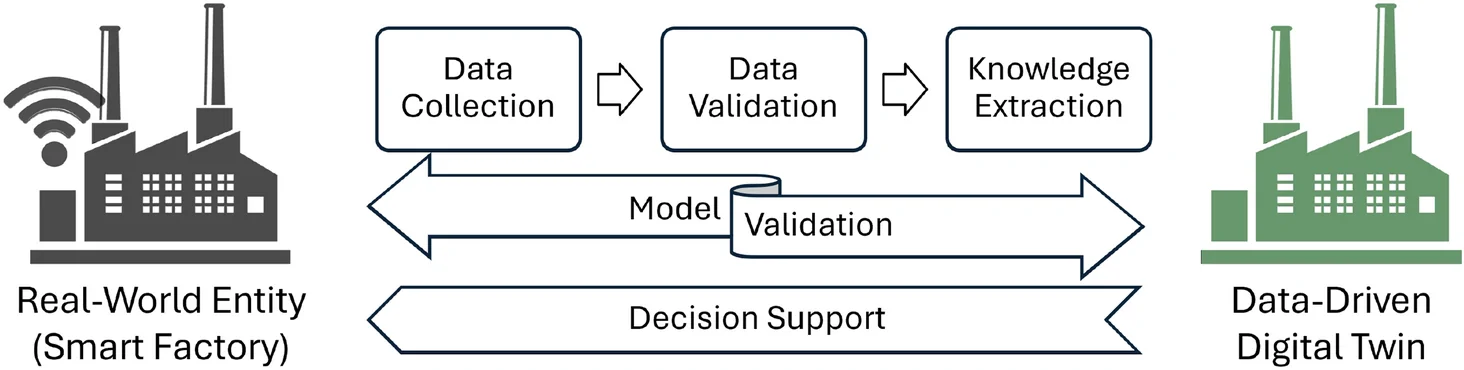
Constituent parts of a digital twin in a manufacturing system^16^.

Although many approaches have been developed to enhance energy efficiency in wastewater treatment plants, issues such as optimizing complex processes, forecasting system behavior under varying conditions, and applying advanced control strategies continue to pose significant challenges. This is where digital twin (DT) technology offers a transformative approach by providing real-time virtual representations of physical systems. The term “digital twin” was originally introduced by Grieves in 2002 at the University of Michigan^15^. As we illustrated in Fig. 1, a DT comprises three essential components. Firstly, DT involves a real-world entity, which could be anything from a single machine to a complete factory. Secondly, DT contains a data-driven simulation model which includes algorithms that define the simulation, along with machine learning and data mining techniques to extract the model from data, and the software that implements these algorithms. Additionally, DT encompasses connectivity elements such as IoT (Internet of Things) systems. Lastly, the DT incorporates data that the real-world entity has produced or is producing. Data is a critical aspect that significantly affects the achievement of the digital modeling goal effectiveness^9^.

DT can be also utilized for monitoring and improving energy efficiency in industrial systems, offering significant potential for optimization. DTs actively assist industries in understanding and modeling the energy consumption behavior of systems, analyzing the system regarding different scenarios, optimizing processes to reduce energy consumption, and predicting and comparing the impacts of each change before implementation in the real system. Although DTs are extensively utilized in numerous fields, research specifically addressing their role in energy management within different industrial systems is still not widespread. As a result, we undertook a comprehensive literature review centered on the application of simulation techniques for improving energy efficiency in industrial systems. Generally, simulation models can be constructed following one of these approaches: discrete event simulation (DES), agent-based simulation (ABS), continuous or system dynamics (SD), and hybrid simulation (HS). Among these approaches, System Dynamics (SD), contrary to DES technique, stands out for its ability to model continuous processes and dynamic behaviors over time, making it particularly effective for analyzing complex, evolving industrial systems^17^.

These models are often used to simulate and enhance the energy efficiency of the processes that evolve continuously, such as chemical reactions in a plant or the flow of materials in a manufacturing process. A recent review by Wang et al.^18^ synthesizes the current state of digital twin applications in wastewater treatment technology, highlighting their potential to enhance operational efficiency, predictive maintenance, and decision-making across various facets of WWTPs and sewage networks. Their work underscores the growing interest in integrating digital twins into wastewater infrastructure, providing a comprehensive overview of existing research and applications. In a related study, numerical modeling combined with real-time monitoring was applied to optimize the aeration system in a wastewater treatment plant, achieving an estimated 20% improvement in energy efficiency. The study demonstrated that predictive modeling and dynamic adjustment strategies can significantly enhance energy management and support more effective plant operation planning^19^. Harrou et al.^20^ conducted a study focused on developing short-term forecasting models for energy consumption in wastewater treatment plants using data-driven soft sensors. They compared traditional time-series methods with deep learning approaches. The results showed that adaptive models enhance the accuracy of energy consumption forecasts. This improvement supports more effective operational and environmental planning. Canete et al.^21^ proposed an adaptive neural-network-based soft sensor to predict effluent characteristics in wastewater treatment, coupled with a genetic algorithm for optimized control. The approach was tested on a large-scale activated sludge simulation model. It demonstrated reduced operational costs, compliance with legal effluent constraints, and potential for real-time implementation with improved control over biological processes.

The SD has also been applied to model and manage waste treatment systems, focusing on the interaction of economic, social, and environmental variables. For example, a hybrid framework combining SD with multi-objective optimization has been developed to enhance decision-making in waste-to-energy management^22^. It achieved significant environmental and financial improvements over traditional simulation approaches. Cheng et al.^23^ proposed a SD model to evaluate the economic potential of using landfill gas for waste leachate evaporation at a regional incineration plant in Chengdu, China. By comparing two scenarios with varying leachate evaporation and landfill gas utilization ratios, they identified an optimal scenario that improved economic performance by 35%. This scenario offered a promising alternative treatment method with potential environmental benefits. However, the model’s limitations, such as unaddressed environmental impacts and economic assumptions, were acknowledged. Another work developed an SD model to optimize municipal solid waste management by analyzing resource use, waste treatment options, and cost structures^24^. The findings suggest that increasing waste sorting efforts and operational productivity can lead to improved financial outcomes. This adaptable model offers valuable insights for decision-making in diverse regions, as demonstrated through a case study in Brazil, where it helped reduce reliance on landfills.

Digital twin technology has been applied to simulate energy consumption and flow in simplified systems. However, its potential extends to modeling energy flow in closed-loop control systems, offering substantial opportunities for advancement. With this, it can support the optimization of wastewater sludge management through thermal treatment methods. These involve the sludge incineration, which is a well-established technology. With its major advantages as the significant reduction in the input sludge volume, and the destruction of toxic organic compounds and pathogens due to high process temperature, as well as odour minimization^25,26^ is considered a good option, despite the high costs it entails to ensure an effective and environmentally safe combustion process. These consist of the energy-expensive costs of dewatering, drying and auxiliary fuel^27^ and high costs that have to be incurred for the efficient exhaust gas purification systems^28^. Since sewage sludge management and processing is said to contribute up to 50% of the total cost of wastewater treatment^28^, an increased drive has been observed to reduce the energy consumption through sludge energy recovery within the system for its drying or for generating electricity^29,30^. Chang et al.^31^ reported that, as far as the incineration of dried sludge is considered, the recovered energy is close to the amount of energy used in the process, although the authors pointed out that available data on energy consumption and recovery are limited.

Guided by the comprehensive overview of the state of the art, to our best knowledge, sludge incineration as a widespread technology, but demanding in terms of specific process requirements, has not lived up to the DT based optimization models. Recently, Song et al.^32^ considered smoldering as a potential technology for high-water content sewage sludge treatment due to low energy consumption and cost, as well as simple operation. They developed a digital twin system based on the back propagation neural network model to predict the characteristics of sewage sludge smoldering, while aiming at improving process velocity, and reducing the concentrations of CO/NOx emissions. Though the authors obtained a satisfactory agreement between predictions and experimental data, they pointed out to the method limitations. These include the necessity of developing a hybrid modeling approach, integrating a universal smoldering mechanism with the data-driven method to preserve data training efficiency, as well as the need for adjustment and optimization of the parameters when scaling up the system.

It should be noted that, depending on the degree of data integration between physical and digital entities, the literature distinguishes digital twins into three subcategories: digital model, digital shadow and digital twin. In this classification, a Digital Model relies on manual data exchange, a Digital Shadow enables automated one-way data flow from the physical system to the digital representation, and a digital twin requires bidirectional integration between the digital and physical domain^33^. This interpretation is consistent with Torfs et al.^34^, who emphasize that a digital twin should maintain an automated live connection to the real entity and evolve continuously with it over time.

In this study, we present a data-informed digital shadow framework integrated with analytical solutions developed using in-house codes. This model is designed to simulate energy consumption in a closed-loop control system, specifically tailored for wastewater treatment facilities, including the sewage sludge incineration, which is of interest in the present study. Given that the system is continuous, we implemented system dynamics (SD) to model the processes accurately. One of the key challenges when simulating a coupled continuous system is accounting for circular dependencies to avoid system lock-ups. Our approach directly addresses this issue, marking a significant step forward in the practical deployment of plant-informed simulation environments. This study presents a novel application of a digital-shadow-based framework for dynamic system analysis of a wastewater treatment subsystem. Unlike traditional modeling, the digital twin framework developed in this work allows for dynamic plant-informed simulation of the physical system. By using operational plant data and simulating various operational scenarios, the DT method shows how different parameters (biogas flow as auxiliary fuel, moisture content in the sludge, combustion air temperature and flow) influence WWTP sludge incineration system performance. This information helps with guiding decision-making toward sustainable configurations, higher efficiency and cost savings. The novelty lies not only in the integration of digital shadow within a closed-loop WWTP system but also in the demonstration of how digital simulations can actively drive system improvements, enhancing operational efficiency, resource recovery, and environmental compliance.

## Description of a closed-loop incineration system of WWTP

This case study builds upon the findings of the previous study detailed in paper^35^, extending the research and applying its methodologies to the incineration system operated in the wastewater treatment plant, which processes an average of 4000 kg/h of dewatered sludge. Initially, raw wastewater undergoes screening and homogenization, followed by aerobic and anaerobic bio-treatment to eliminate contaminants. Subsequently, the water treatment process involves mechanical dewatering and compaction of sewage sludge (SS) using membrane presses and centrifuges, before entering the incineration system.

In the present study, the focus is on the sewage sludge incineration system of the WWTP. It must comply with European standards that are regulated under Directive 2010/75/UE on Industrial Emissions (modernized version of Directive 2010/76/UE), which defines strict technical requirements, operational conditions and emission limits for waste incineration, in accordance with the BAT (best available techniques) guidelines. This primarily includes the incineration temperature, which is regulated to a minimum of 850$\mathrm {^o}$C (and must be maintained for at least 2 seconds) to ensure the destruction of hazardous compounds (dioxins and furans).

**Fig. 2:**
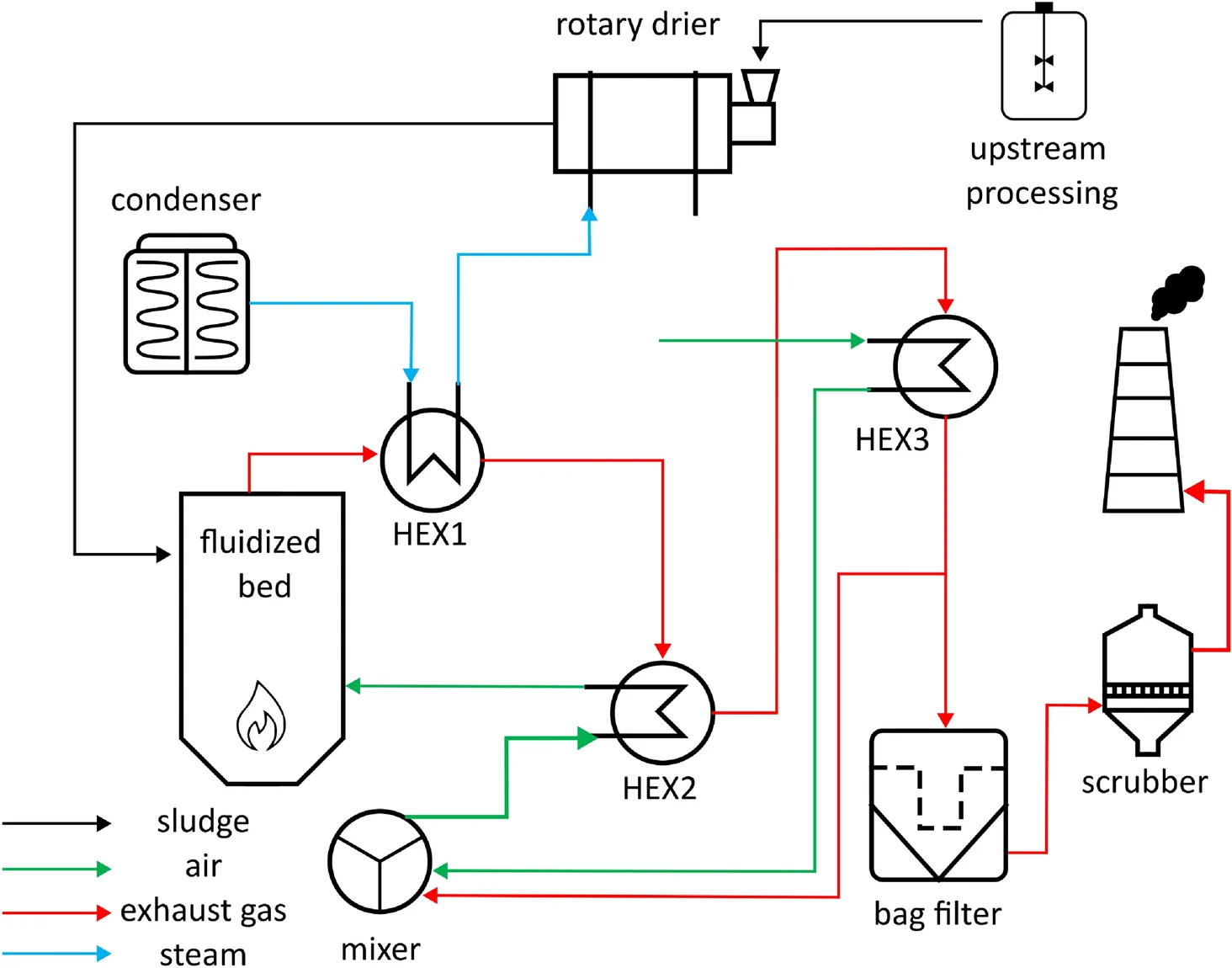
Simplified diagram of the analyzed sewage sludge incineration system of the WWTP.

A simplified configuration of the sewage sludge incineration system of the WWTP is considered to investigate the energy consumption of the system (Fig. 2). It comprises a rotary dryer, a fluidized bed furnace (FBF), a condenser, a bag filter, a scrubber, a mixer, and three heat exchangers (HEX1, HEX2, HEX3). The process begins with the super-heated steam in the rotary dryer absorbing moisture from the sludge. The pre-dried sludge is incinerated in the FBF, and the resulting fumes are used to heat steam in HEX1. Excess steam is then expelled to the condenser. Airflow, temperature, and composition are regulated by a mixer before entering HEX2. The flue gas is subsequently routed to HEX3 to preheat the combustion air for the fluidized bed, and the exhaust heats the ambient air in HEX3 before passing through the bag filter and scrubber, reducing hazardous material concentrations before release into the environment. Biogas generated in the anaerobic digestion part of a wastewater treatment plant is injected into the fluidized bed when bed temperature drops. This drop is typically caused by high moisture content or operational fluctuations, and the biogas injection helps to stabilize combustion.

## Methodology

**Fig. 3:**
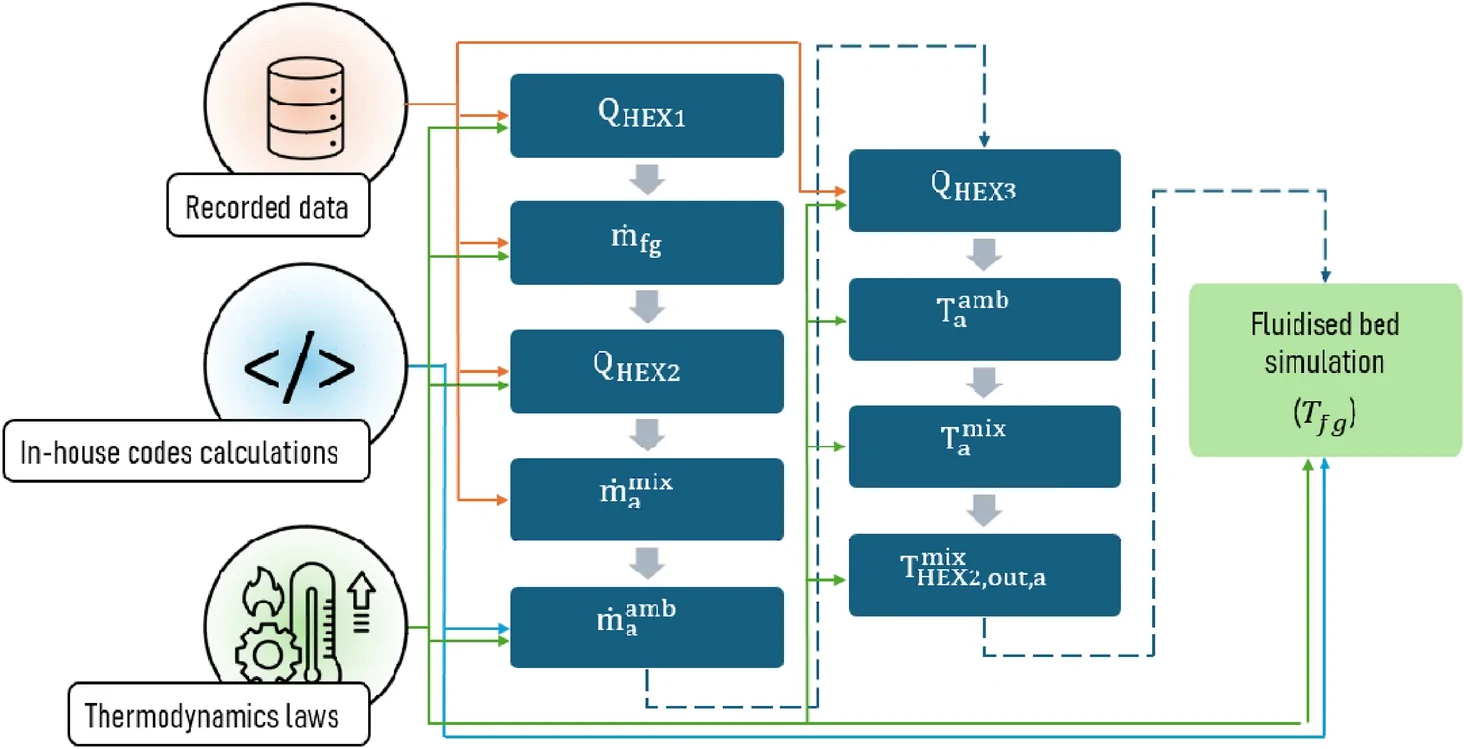
Graphical explanation of the performed analysis.

The methodology involves a multi-step process, incorporating data collection, analytical calculations using in-house codes written in Fortran and Python, and simulation modeling in AnyLogic. Schematic explanation of the procedure is presented in Fig. 3. In the first step, analytical calculations of flue gas composition and temperature of combustion were conducted. Then, the obtained information, together with the gathered measurement data were used for the further calculations. The simulation of the wastewater treatment plant was conducted using AnyLogic software, with the main objective of predicting the reactor temperature over a 24-h period. The model was then validated with the use of the real data gathered in the system of the wastewater treatment plant. The system operating data included the sludge flow rate, biogas flow rate, sludge moisture content, air temperature, steam temperature at the inlet and outlet of heat exchanger No. 3, temperatures of flue gases at the inlets and outlets of heat exchangers 1, 2 and 3. The available dataset covered a 24-h period only, as access to longer-term operational records was restricted by data-access and security constraints associated with the wastewater treatment plant. Although a longer dataset would enable a more comprehensive assessment under a wider range of operating conditions, the present study uses the available data as an initial basis for model development and proof-of-concept validation.

### Analytical calculations

In addition to the acquired data, it was necessary to estimate additional parameters, such as flue gas composition, and combustion temperature, and their influence on the furnace temperature. In the first step, the gas composition and combustion temperature were calculated for three moisture contents of sludge (20%, 30%, and 40%) and three recirculation rates (20%, 30%, and 40%). For each combination of moisture and recirculation rate, the analysis was performed for ten *λ* (excess-air ratio) values ranging from 1.14 up to 2.38. Then, based on the obtained data, a linear regression model was used in order to estimate a function for the furnace temperature.

#### Flue gas stream: chemical calculations

To determine the amount of flue gas and composition, chemical calculations were performed considering a global reaction of combustion of sewage sludge. A general empirical formula of $\mathrm {C_x H_y O_z N_q}$ for the sludge was assumed with $\textrm{x}$, $\textrm{y}$, $\textrm{z}$, $\textrm{q}$ defined based on the chemical sludge composition. The combustion with air excess was taken into account with the resulting flue gas composed of the basic compounds such as $\mathrm {CO_2}$, $\mathrm {H_2O}$, $\mathrm {O_2}$ and $\mathrm {N_2}$, according to the reaction:

1 $$\begin{aligned} \mathrm {C_x H_y O_z N_q} + n_\mathrm{{ox}} \lambda (\mathrm {O_2+3.76N_2}) \rightarrow n_\mathrm{{CO_2}} \mathrm {CO_2} + n_\mathrm{{H_{2}O}} \mathrm {H_{2}O} + n_\mathrm{{O_2}} \mathrm {O_2} + n_\mathrm{{N_2}} \mathrm {N_2}, \end{aligned}$$

where *n* denotes the number of moles and *λ* is the excess air ratio which is expressed by:

2 $$\begin{aligned} \lambda =\frac{1}{AFR} \frac{m_\mathrm{{ox}}}{m_\mathrm{{ss},d}}. \end{aligned}$$

In the above, *AFR* represents air-to-fuel ratio, $m_\mathrm{{ox}}$ and ${m_\mathrm{{ss,d}}}$ stand for the mass of oxidizer and dry sewage sludge, respectively. The resulting flow rate of flue gases $\dot{m}_\mathrm{{fg}}$ was determined from the mass balance equation:

3 $$\begin{aligned} \dot{m}_\mathrm{{ss}}^{in}+\dot{m}_\mathrm{{ox}} =\dot{m}_\mathrm{{fg}}+\dot{m}_\mathrm{{a}}, \, \end{aligned}$$

where $\dot{m}_\textrm{a}$ represents the mass rate of ash.

Knowing the chemical composition of the fuel (sludge), based on the above equations, the amount of $\mathrm {CO_2}$, $\mathrm {H_2O}$, $\mathrm {O_2}$, and $\mathrm {N_2}$, and the flue gas stream were calculated for various configurations of moisture, recirculation rate and excess air ratio.

#### Combustion temperature

In the sewage sludge combustion system, part of the exhaust gas is recirculated back to the combustion chamber. This is mainly to control the process temperature and keep it at the required level that prevents $\mathrm {NO_x}$ emissions. To simulate the process with flue gas recirculation the computation method involved two steps. In the first one, the combustion temperature for the process stage with no recirculation was determined as the initial process temperature for the next step. Here, the energy balance for the furnace chamber includes the input energy of: (i) dry sewage sludge ($\dot{Q}_\mathrm{{ss,d}}$), (ii) moisture contained in the sludge ($\dot{Q}_\mathrm{{H_2O}}$), (iii) oxidizer ($\dot{Q}_\mathrm{{ox}}$) and (iv) sludge chemical energy ($\dot{Q}_\mathrm{{ch}}$), and the outflow thermal energy of: (v) flue gas ($\dot{Q}_\textrm{fg}$), (vi) steam ($\dot{Q}_\mathrm{{H_2O}}^\mathrm{{fg}}$) and (vii) removed ash ($\dot{Q}_\mathrm{{a}}$), and is written as

4 $$\begin{aligned} \dot{Q}_\mathrm{{ss,d}} + \dot{Q}_\mathrm{{ch}} + \dot{Q}_\mathrm{{H_2O}} + \dot{Q}_\mathrm{{ox}} = \dot{Q}_\mathrm{{fg}} + \dot{Q}_\mathrm{{H_2O}}^\mathrm{{fg}} +\dot{Q}_\mathrm{{a}}, \ \end{aligned}$$

where

5 $$\begin{aligned} \dot{Q}_\mathrm{{}ss,d}&= \left( \dot{m} c \right) _\mathrm{{ss,d}} T^{in}, \, \end{aligned}$$

6 $$\begin{aligned} \dot{Q}_\mathrm{{ch}}&= \dot{m}_\mathrm{{ss}} \textrm{HHV}, \, \end{aligned}$$

7 $$\begin{aligned} \dot{Q}_\mathrm{{H_2O}}&= Y_\mathrm{{H_2O}} \dot{m}_\mathrm{{ss}} h_\mathrm{{H_2O}}(T^{in}), \, \end{aligned}$$

8 $$\begin{aligned} \dot{Q}_\mathrm{{ox}}&= \dot{m}_\mathrm{{ox}} \sum _i Y_{i,\textrm{ox}}h_{i}(T_\mathrm{{ox}}), \, \end{aligned}$$

9 $$\begin{aligned} \dot{Q}_\mathrm{{fg}}&= \dot{m}_\mathrm{{fg}} \sum _i Y_{i,\textrm{fg}}h_{i}(T_\mathrm{{fg}}), \, \end{aligned}$$

10 $$\begin{aligned} \dot{Q}_\mathrm{{H_2O}}^\mathrm{{fg}}&= Y_\mathrm{{H_2O}} \dot{m}_\mathrm{{ss}} h_\mathrm{{H_2O}}(T_\mathrm{{fg}}), \, \end{aligned}$$

11 $$\begin{aligned} \dot{Q}_\mathrm{{a}}&= \left( 1-Y_\mathrm{{H_2O}} \right) \dot{m}_\mathrm{{ss}} c_\mathrm{{a}} (T_\mathrm{{fg}}). \, \end{aligned}$$

In the above, $\dot{m}$ stands for the mass flow rate, *c* is the specific heat, whereas *T* represents temperature, *h* is the specific enthalpy, $Y_\mathrm{{H_2O}}$ is the mass fraction of moisture in the sludge at the inlet to the furnace and $Y_i$ is the mass fraction of *i*-th gas component. Subscripts $\textrm{a}$, $\textrm{fg}$, and $\mathrm {H_2O}$ represent ash, flue gas, and water/steam, respectively. The quantity $\textrm{HHV}$ is the higher heating value of the sludge. Superscript *in* stands for the inlet parameter value. The enthalpies of compounds were determined using the *RefProp* database^36^. To calculate the combustion temperature sought, the iteration method was applied.

Based on the parameters obtained at the first step, for the set flow rate of a recirculated flue gas $\dot{m}_\mathrm{{fg}}^r$, the composition of an oxidizing gas mixture supplied to the furnace chamber and the resulting flue gas composition were calculated, according to the system of mass balance equations:

12 $$\begin{aligned} \dot{m}_\mathrm{{ss}}+\dot{m}_\mathrm{{ox}}+\dot{m}_\mathrm{{fg}}^r&=\dot{m}_\mathrm{{fg}}+\dot{m}_\mathrm{{a}}, \end{aligned}$$

13 $$\begin{aligned} \dot{m}_\mathrm{{fg}}&=\dot{m}_\mathrm{{fg}}^{out}+\dot{m}_\mathrm{{fg}}^r, \end{aligned}$$

where *out* refers to the outlet flue gas stream from the combustion system.

The combustion temperature was then recalculated, solving iteratively the modified energy balance for the recirculation-type system, formulated as:

14 $$\begin{aligned} \dot{Q}_\mathrm{{ss,d}} + \dot{Q}_\mathrm{{ch}} + \dot{Q}_\mathrm{{H_2O}} + \dot{Q}_\mathrm{{ox}} + \dot{Q}_\mathrm{{fg}}^{r}=\dot{Q}_\mathrm{{fg}}^{out}+\dot{Q}_\mathrm{{H_2O}}^\mathrm{{fg}} +\dot{Q}_\mathrm{{a}}, \end{aligned}$$

where $\dot{Q}_\mathrm{{fg}}^{r}$ and $\dot{Q}_\mathrm{{fg}}^{out}$ represent the energy of recirculated and the remaining (outlet) flue gas streams, respectively:

15 $$\begin{aligned} \dot{Q}_\mathrm{{fg}}^{r}&= \dot{m}_\mathrm{{fg}}^r \sum _i Y_{i,r}h_{i}(T_\mathrm{{ox}}), \, \end{aligned}$$

16 $$\begin{aligned} \dot{Q}_\mathrm{{fg}}^{out}&= \dot{m}_\mathrm{{fg}}^{out} \sum _i Y_{i,\textrm{fg}}h_{i}(T_\mathrm{{fg}}), \, \end{aligned}$$

which allowed to calculate the temperature of combustion, needed for the next step of the analysis.

Following the stoichiometric calculations, five factors influencing the furnace temperature were established: sludge flow rate ($\dot{m}_\mathrm{{ss}}$), sludge moisture content ($Y_\mathrm{{H_2O}}$), mixed air flow rate ($\dot{m}_\mathrm{{a}}^\mathrm{{mix}}$) and temperature ($T_\mathrm{{a}}^\mathrm{{mix}}$), and the recirculation rate (*R*). The regression model was fitted using 90 data points generated from the analytical thermochemical calculations. The regression structure was selected systematically as a second-order polynomial including linear, quadratic, and two-factor interaction terms in order to capture the nonlinear and coupled effects of the operating variables on furnace temperature. The resulting equation should therefore be interpreted as a computationally efficient metamodel of the analytical thermochemical calculations rather than as a direct first-principles combustion model. Based on this regression structure, an equation for calculating the furnace temperature as a function of these five factors was derived in Python:

17 $$\begin{aligned} \begin{aligned} T_{frn}&= a_1 \cdot \dot{m}_\mathrm{{ss}} +b_1 \cdot \dot{m}_\mathrm{{a}}^\mathrm{{mix}} + c_1 \cdot T_\mathrm{{a}}^\mathrm{{mix}} + d_1 \cdot R + e_1 \cdot Y_\mathrm{{H_2O}} \\&+ a_2 \cdot \dot{m}_\mathrm{{ss}}^2 + b_2 \cdot (\dot{m}_\mathrm{{a}}^\mathrm{{mix}})^2 + c_2 \cdot (T_\mathrm{{a}}^\mathrm{{mix}})^2 + d_2 \cdot R^2 + e_2 \cdot Y_\mathrm{{H_2O}}^2 \\&+ f_1 \cdot \dot{m}_\mathrm{{ss}} \cdot \dot{m}_\mathrm{{a}}^\mathrm{{mix}} + f_2 \cdot \dot{m}_\mathrm{{ss}} \cdot T_\mathrm{{a}}^\mathrm{{mix}} + f_3 \cdot \dot{m}_\mathrm{{ss}} \cdot R + f_4 \cdot \dot{m}_\mathrm{{ss}} \cdot Y_\mathrm{{H_2O}} \\&+ f_5 \cdot \dot{m}_\mathrm{{a}}^\mathrm{{mix}} \cdot T_\mathrm{{a}}^\mathrm{{mix}} + f_6 \cdot \dot{m}_\mathrm{{a}}^\mathrm{{mix}} \cdot R + f_7 \cdot \dot{m}_\mathrm{{a}}^\mathrm{{mix}} \cdot Y_\mathrm{{H_2O}} \\&+ f_8 \cdot T_\mathrm{{a}}^\mathrm{{mix}} \cdot R + f_9 \cdot T_\mathrm{{a}}^\mathrm{{mix}} \cdot Y_\mathrm{{H_2O}} + f_{10} \cdot R \cdot Y_\mathrm{{H_2O}} \end{aligned} \end{aligned}$$

Values of coefficients $a_1 - f_{10}$ can be found in the Appendix. In the further step, the determined function was implemented into the AnyLogic software in order to simulate the wastewater treatment system work in various conditions.

### Dynamic simulation in AnyLogic

Systems of ordinary differential equations and partial differential equations serve as mathematical frameworks, extensively applied in scientific and engineering fields for dynamic system modeling^37,38^. A governing equation describes the relationships among variables, such as temperature or chemical concentration, representing state variables in a dynamic system. The equations are derived from general empirical observations and specific hypotheses, such as the first law of thermodynamics. However, in real-world systems, formulating governing equations becomes challenging due to the complexity of the system and the simultaneous occurrence of multiple phenomena, leading to uncertainties in existing principles. With advancements in sensor technology, it is now possible to sample data from dynamic systems, opening doors for extracting insights into their physical behavior. This has sparked increased interest in recent years in creating data-driven techniques to uncover the governing equations behind these dynamic systems^39^. Comprehending the behavior of dynamic systems through data is an important scientific and practical problem, particularly in engineering where testing many scenarios during prototyping is necessary.

In the present study, the dynamic behavior of the model is represented through time-varying operational data introduced into the AnyLogic simulation over the 24-h period, with recorded plant values updated at 1-min intervals. At each simulation step, the measured inputs are used to update the coupled mass- and energy-balance calculations of the furnace, mixer, dryer, and heat exchangers. Because the system is thermally and operationally interconnected, the calculated state of each component influences the simulation of the overall system at the subsequent time step. In this way, changes in one part of the process propagate through the interconnected components over time. The model therefore represents a dynamically coupled process simulation driven by time-dependent plant data, rather than a fully mechanistic transient model with explicit delay or storage-state equations for each unit.

#### Modeling in AnyLogic

**Fig. 4:**
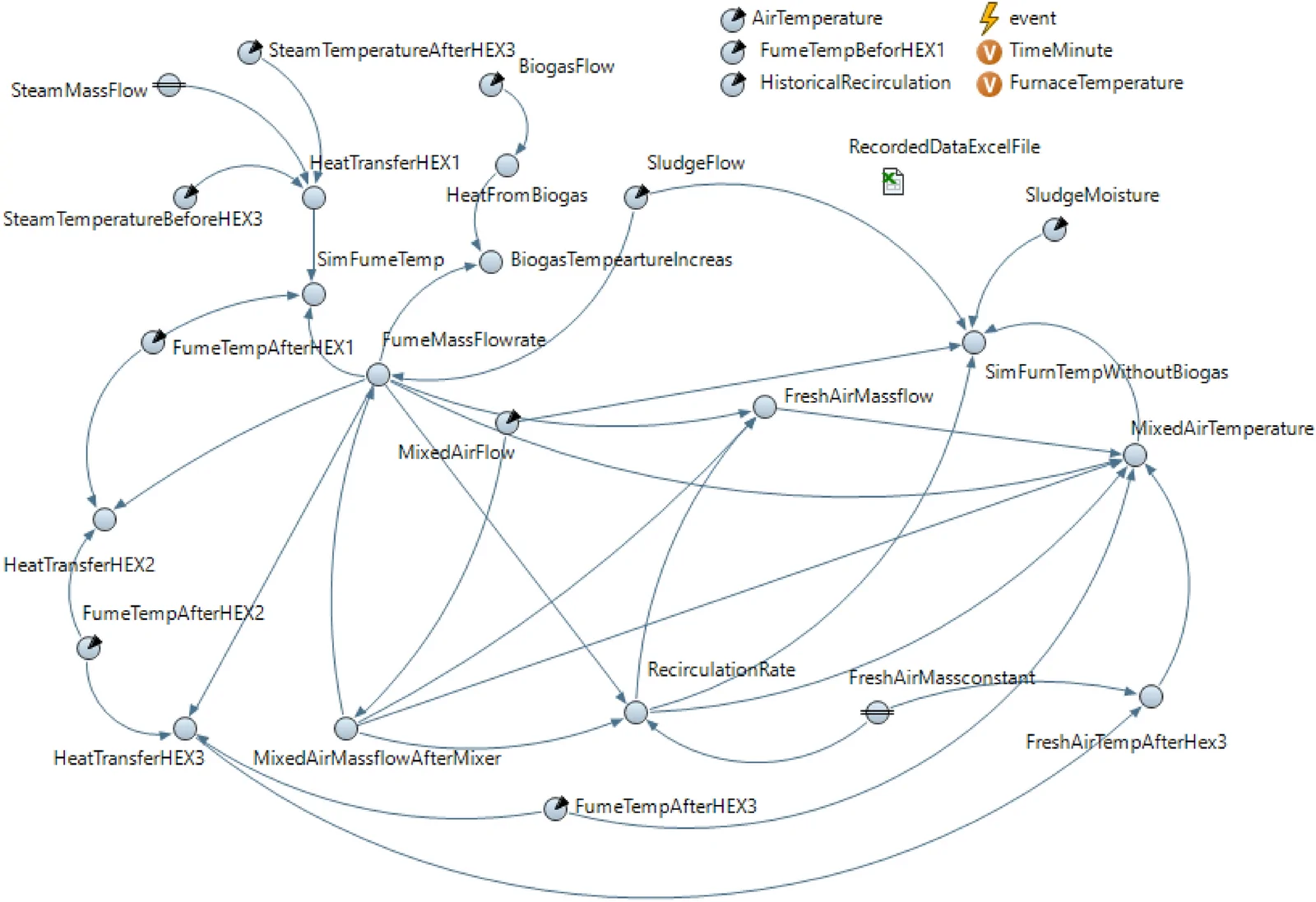
Diagram of the simulation procedure.

The procedure of the simulation is presented on the scheme in Fig. 4. To initiate the modeling process in AnyLogic, our primary objective is to determine the heat transfer occurring within heat exchanger 1 (HEX1). This involves calculating the mass flow rate of steam entering the heat exchanger. Leveraging recorded data for the inlet and outlet temperatures of HEX1, we can compute the heat transferred between the flue gas and steam ($\dot{Q}_\mathrm{{HEX1}}$) utilizing the equation:

18 $$\begin{aligned} \dot{Q}_\mathrm{{HEX1}}=\dot{m}_\mathrm{{st}} \cdot \left( h_\mathrm{{st}}^{out}-h_\mathrm{{st}}^{in}\right) , \end{aligned}$$

where $\dot{m}_\mathrm{{st}}$ denotes the mass flow rate of steam, and $h^{in}_\mathrm{{st}}$ and $h^{out}_\mathrm{{st}}$ represent the enthalpies of the steam at the inlet and outlet of HEX1, respectively.

To model the enthalpy of steam within the simulation, a linear correlation has been established based on the properties of water at atmospheric pressure *h=2.0514 T+2465.7*^40^. The assumption of negligible pressure drop is considered in this correlation. It allows for a simplified yet accurate estimation of enthalpy values without the need for direct table lookup.

In the subsequent step, the mass flow rate of the flue gas is determined by utilising the heat transfer in the heat exchanger. Assuming a constant specific heat for the flue gas ($c_{p,\textrm{fg}}$), and considering the temperature differences between the outlet and inlet of the flue gas, the mass flow rate of the flue gas can be calculated from:

19 $$\begin{aligned} \dot{m}_\mathrm{{fg}}=\frac{\dot{Q}_\mathrm{{HEX1}}}{c_{p,\textrm{fg}}\left( T^{out}_\mathrm{{fg}}-T^{in}_\mathrm{{fg}}\right) } \end{aligned}$$

Moving to the next stage in HEX2, heat transferred in heat exchanger 2 is determined by utilising the previously obtained mass flow rate of the flue gas. By considering the temperatures of the flue gas at the inlet and outlet of HEX2, the heat flow can be calculated using the equation:

20 $$\begin{aligned} \dot{Q}_\mathrm{{HEX2}}=\dot{m}_\mathrm{{fg}} c_{p,\textrm{fg}} \left( T_\mathrm{{fg},\textrm{HEX2}}^{out}-T_\mathrm{{fg},\textrm{HEX2}}^{in}\right) \end{aligned}$$

Next, the mass flow of the mixed air passing through HEX2 and entering the furnace is determined. Initially, the volume flow rate of the mixed air is recorded. To obtain the density of the mixed air, an assumption is made that the temperature of the mixed air falls within the range of 200 to 300 $^\circ$C. With this assumption, the dynamic parameter of mass flow is obtained by multiplying the density of the mixed air with the recorded volume flow rate:

21 $$\begin{aligned} \dot{m}_\mathrm{{a}}^\mathrm{{mix}}=\rho _\mathrm{{a}}^\mathrm{{mix}} \dot{V}_\mathrm{{a}}^\mathrm{{mix}}. \end{aligned}$$

Within the mixer, a portion of the flue gas from HEX3 is combined with the ambient air preheated in HEX3, forming the mixed air stream that enters HEX2. With the known mass flow rates of the mixed air and the flue gas, and considering the recirculation percentage of the flue gas obtained through analytical solution employing the mass conservative equation, the mass flow rate of the ambient air can be determined from the relationship:

22 $$\begin{aligned} \dot{m}_\mathrm{{a}}^\mathrm{{amb}}=\dot{m}_\mathrm{{a}}^\mathrm{{mix}}-\left( \frac{R}{100} \cdot \dot{m}_\mathrm{{fg}}\right) . \end{aligned}$$

Further, the temperature of the supplied air after HEX3 is determined using the equation:

23 $$\begin{aligned} T_\mathrm{{a},\textrm{HEX3}}^\mathrm{{amb}}=T_\mathrm{{amb}}+\frac{\dot{Q}_\mathrm{{HEX3}}}{\dot{m}_\mathrm{{a}}^\mathrm{{amb}} \cdot c_{p,\textrm{a}}}, \end{aligned}$$

utilizing the thermal output of HEX3 calculated for the known mass flow rate of the flue gas and the inlet and outlet temperatures, given as:

24 $$\begin{aligned} \dot{Q}_\mathrm{{HEX3}}=\dot{m}_\mathrm{{fg}} c_{p,\textrm{fg}} \left( T_\mathrm{{fg},\textrm{HEX3}}^{in}-T_\mathrm{{fg},\textrm{HEX3}}^{out}\right) , \end{aligned}$$

and assuming the ambient temperature ($T_\mathrm{{amb}}$) of the air in September at the water treatment plant location is approximately 14$^\circ$ C.

When returning to the mixer, the temperature of the mixed air entering HEX2 is determined. With the known mass flow rate of the mixed air, the mass flow rate of the preheated ambient air along with its temperature after HEX3, the mass flow rate of the flue gas, and the recirculation percentage, energy conservation in the mixer allows for the calculation of the temperature of the mixed air using the equation:

25 $$\begin{aligned} \begin{aligned} \dot{m}_\mathrm{{a}}^\mathrm{{mix}} \cdot c_{p,\textrm{a}}^\mathrm{{mix}} \cdot T_\mathrm{{a}}^\mathrm{{mix}}=\dot{m}_\mathrm{{a}}^\mathrm{{amb}} \cdot c_{p,\textrm{a}} \cdot T_\mathrm{{a}, HEX3}^\mathrm{{amb}}+\\ \left( 1-\frac{R}{100}\right) \cdot \dot{m}_\mathrm{{fg}} \cdot c_{p,\textrm{fg}} \cdot T_\mathrm{{fg}}^{out,\textrm{mix}} \end{aligned} \end{aligned}$$

Moving forward to HEX2, the temperature of the mixed air exiting HEX2 and directed to the furnace is determined. Utilising the heat transferred in HEX2, along with the known mass flow rate of the mixed air and its temperature at the inlet of HEX2, the temperature at the outlet of HEX2 can be calculated using the equation:

26 $$\begin{aligned} T_\mathrm{{a},\textrm{HEX2}}^{out,\textrm{mix}}=T_{a,\textrm{HEX2}}^{in,\textrm{mix}}+\frac{\dot{Q}_\mathrm{{HEX2}}}{\dot{m}_\mathrm{{a}}^\mathrm{{mix}}\cdot c_{p,\textrm{a}}^\mathrm{{mix}}} \end{aligned}$$

Finally, as described in the previous subsection, the linear regression analysis of analytical results was performed and utilised. This regression model takes into account key parameters, such as the mass of sludge and moisture, recirculation percentage, air flow rate, air temperature, and the amount of biogas. By inputting these functions into the regression model, we obtain an estimated temperature of the flue gas.

## Results and discussion

### Model validation

**Fig. 5:**
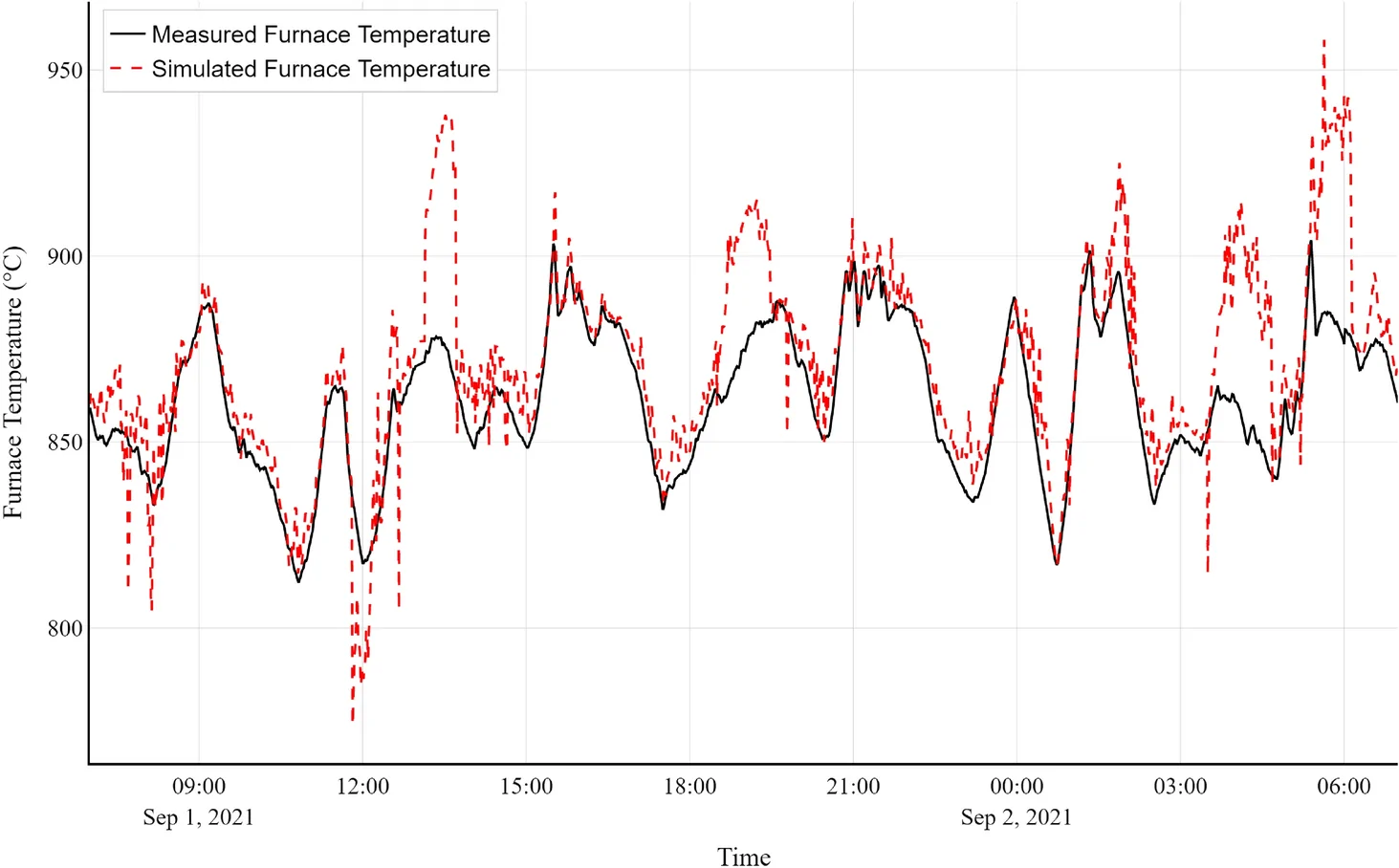
Comparison of measured and simulated reactor temperature.

In our validation process, we meticulously compared the simulated furnace temperature with historical sensor data, specifically focusing on observations from the 1st of September 2021, as depicted in Fig. 5. The analysis is based on a 24-h dataset. It includes daily fluctuations in inflow rate, temperature, and sludge characteristics. These short-term dynamics are critical for evaluating the immediate responsiveness and stability of the simulation model. Although access to longer-term operational data was limited by plant data restrictions, we acknowledge that an extended dataset would enable a more comprehensive validation across varying operational scenarios, such as seasonal changes or maintenance cycles. Nonetheless, the 24-h data serves as an initial basis for validating the short-term dynamic behavior of the model. By running the simulation model under conditions mirroring those of the historical period, we sought to ensure its accuracy in replicating real-world dynamics.

The comparison revealed a reasonable alignment between the simulated and measured reactor temperatures, indicating that our simulation model captures the essential behavior of the system. This was supported by quantitative metrics such as mean squared error (MSE), mean absolute error (MAE), and the coefficient of determination (R-squared). With an MSE of 331.53, an MAE of 11.78, and an R-squared value of 0.71, the model was able to reasonably reproduce the observed trends and provided meaningful insights into the system’s dynamics. The most noticeable deviations, particularly around 04:00, 06:00, 13:00, and 19:00, coincide with intervals of biogas injection into the furnace. These periods are associated with rapid thermal adjustments in the system, which the current model captures only approximately. This suggests that the dynamic response to auxiliary fuel injection could be represented more accurately in future work. While not fully capturing all variations, this level of correspondence suggests that the simulation offers a useful first-order representation of the system for the purposes of analysis and proof-of-concept assessment.

### Furnace output

**Fig. 6:**
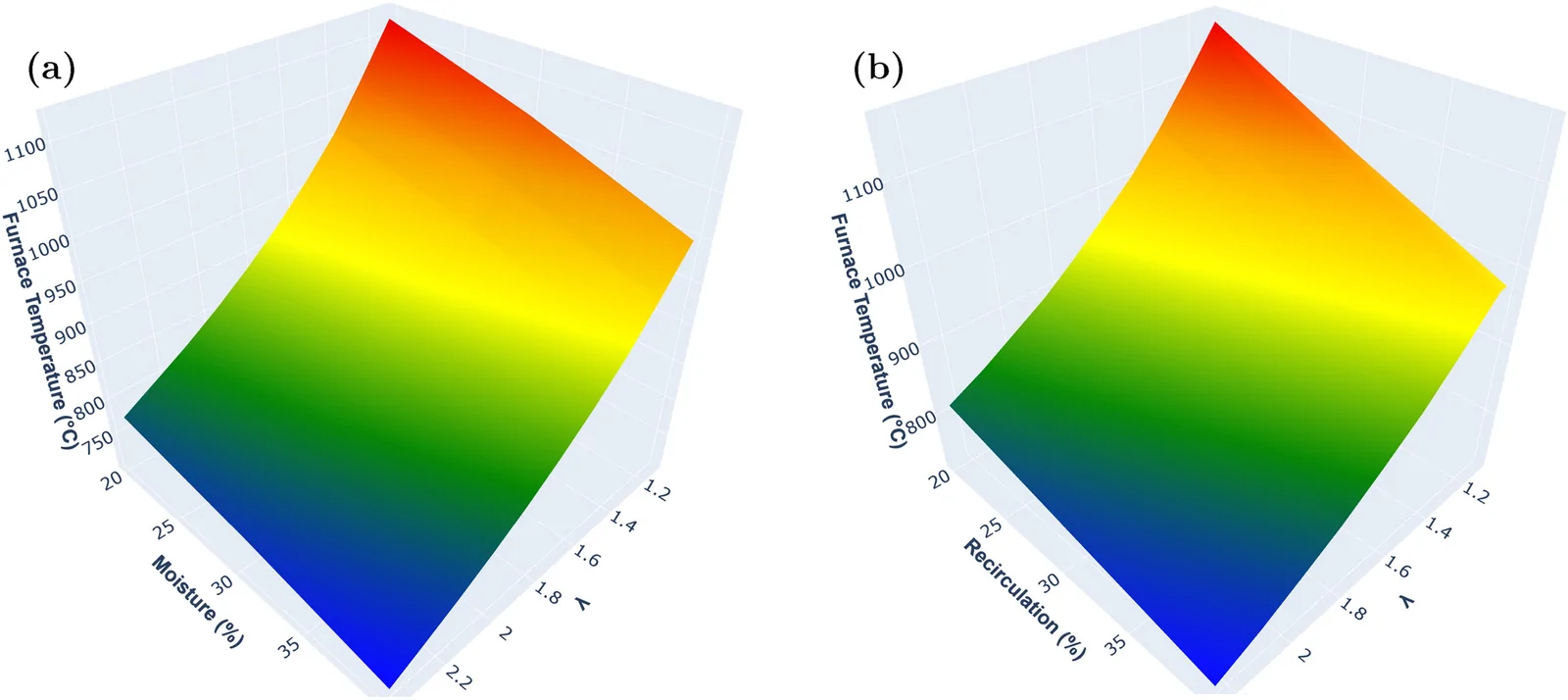
Furnace temperature under different conditions: (**a**) influence of moisture content and excess air ratio (recirculation rate 30*%*), (**b**) influence of recirculation rate and excess air ratio (moisture content 30*%*); Sludge flow 4 tons/h.

**Fig. 7:**
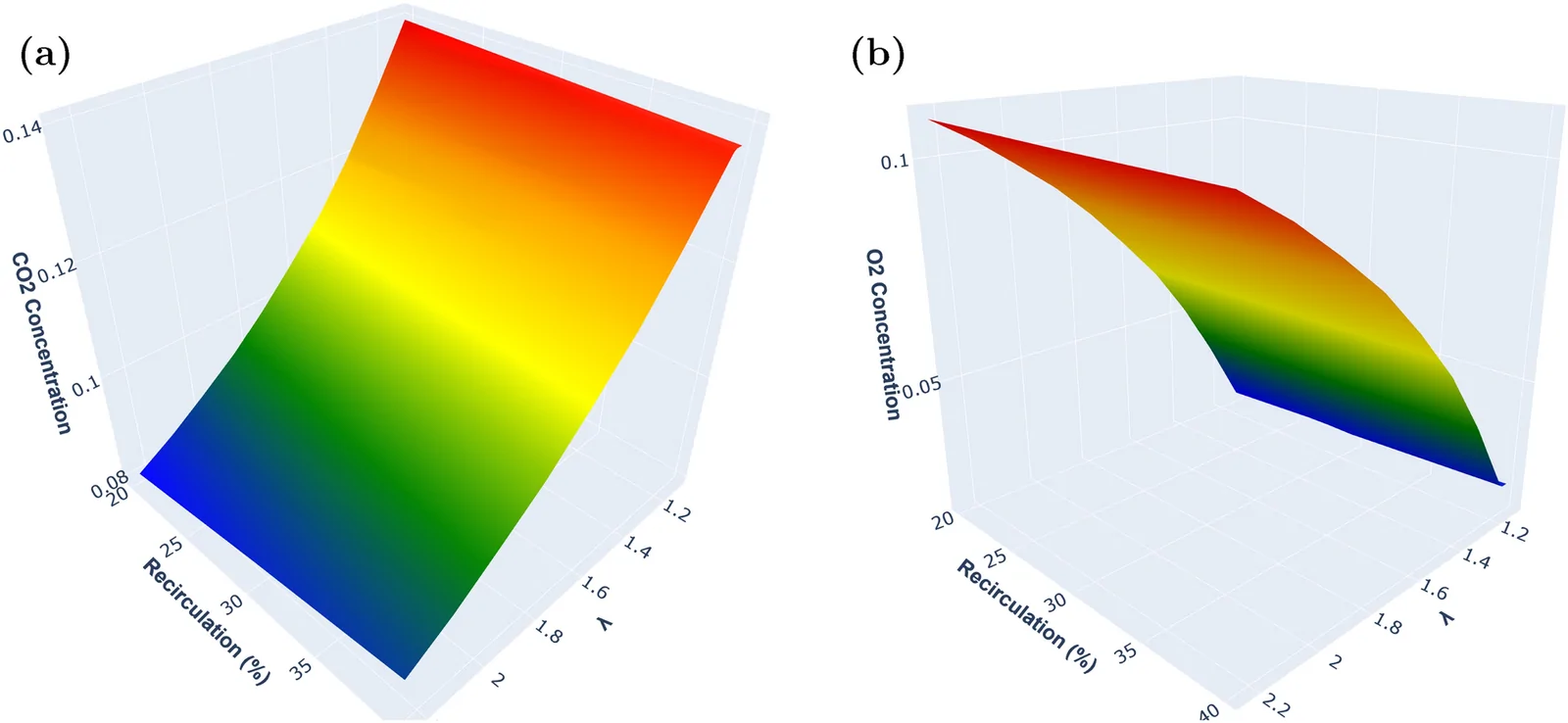
Influence of recirculation rate and excess air ratio on: (**a**) CO_2_ concentration, (**b**) O_2_ concentration; (Operating conditions: sludge flow 4 tons/h, moisture content 30%).

**Fig. 8:**
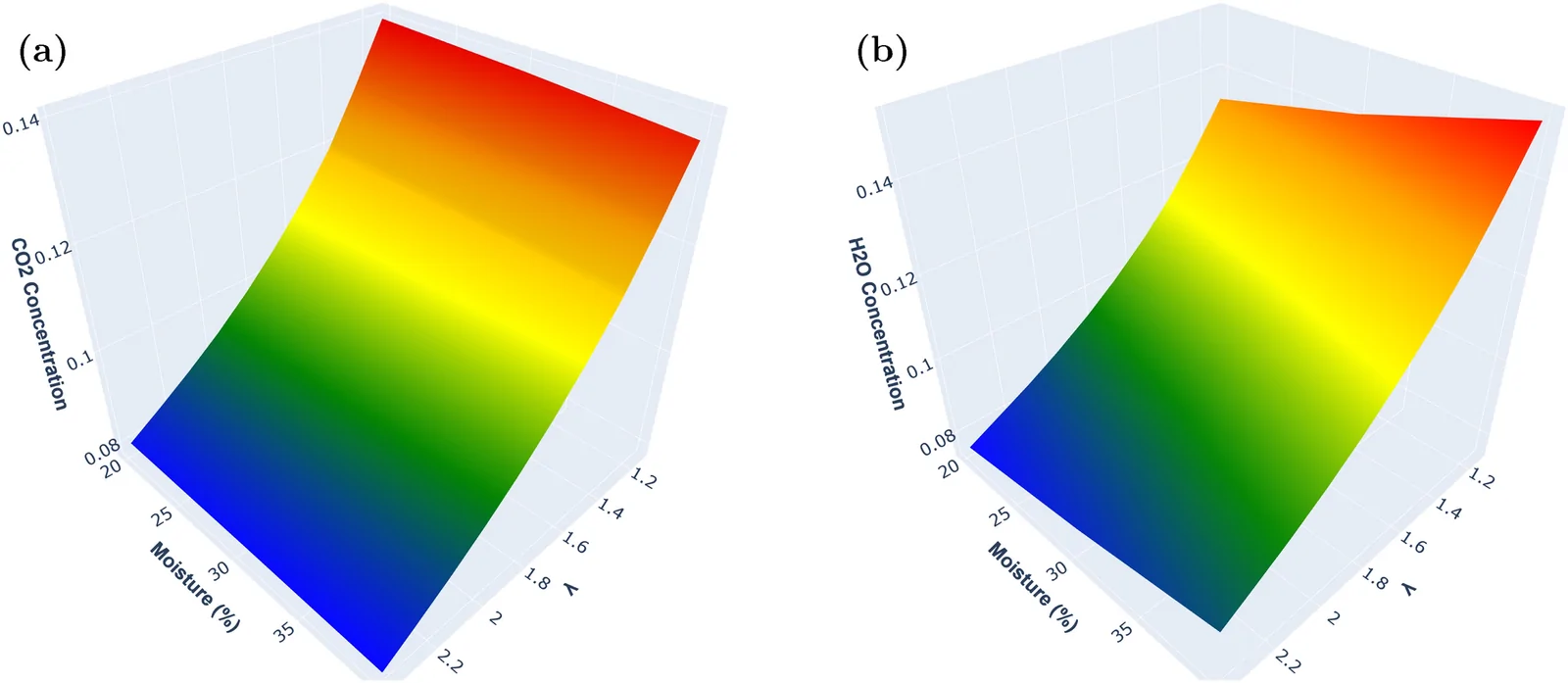
Influence of moisture content and excess air ratio on: (**a**) CO_2_ concentration, (**b**) H_2_O concentration; (operating conditions: sludge flow 4 tons/h, recirculation rate 30%).

Figures 6, 7 and 8 show relations between various parameters of the fluidized bed furnace. Furnace temperature in relation to excess air fuel ratio is shown in Fig. 6. Obviously, the decrease in the excess air ratio is inseparably connected with the temperature increase. The two plots, however, show a slight variation in temperature with the change in moisture content (Fig. 6a) and the recirculation rate (Fig. 6b) for a given excess air ratio. In the first case, representing the case of a recirculation rate at the level of 30%, the decrease in moisture content from 40 to 20% corresponds to a slight temperature increase (of about 30 degrees) for a high excess air ratio (of 2.2). With a decreasing excess air ratio, the decreasing moisture causes even more temperature growth. The influence of the recirculation rate is very similar. Recirculation decreasing from 40 to 20% results in a slight increase in temperature. This effect is more pronounced at lower excess air ratios.

Figure 7 shows the influence of recirculation rate and excess air ratio on $\mathrm {CO_2}$ and $\mathrm {O_2}$ concentration. As expected, $\mathrm {CO_2}$ concentration (Fig. 7a) is increasing with the decrease in the excess air ratio. Simultaneously, $\mathrm {O_2}$ concentration (Fig. 7b) is increasing with an increase in the excess air ratio. Recirculation seems to have a very small or no influence on both $\mathrm {CO_2}$ and $\mathrm {O_2}$ concentrations in the investigated range.

Finally, Fig. 8 displays the influence of moisture and excess air ratio on $\mathrm {CO_2}$ and $\mathrm {H_2O}$ concentrations. It seems that moisture has either a very small or no influence on the $\mathrm {CO_2}$ concentration (Fig. 8a). However, when it comes to $\mathrm {H_2O}$ concentration (Fig. 8b), it obviously increases with an increase in the moisture content in the sludge.

### Scenario simulation

**Fig. 9:**
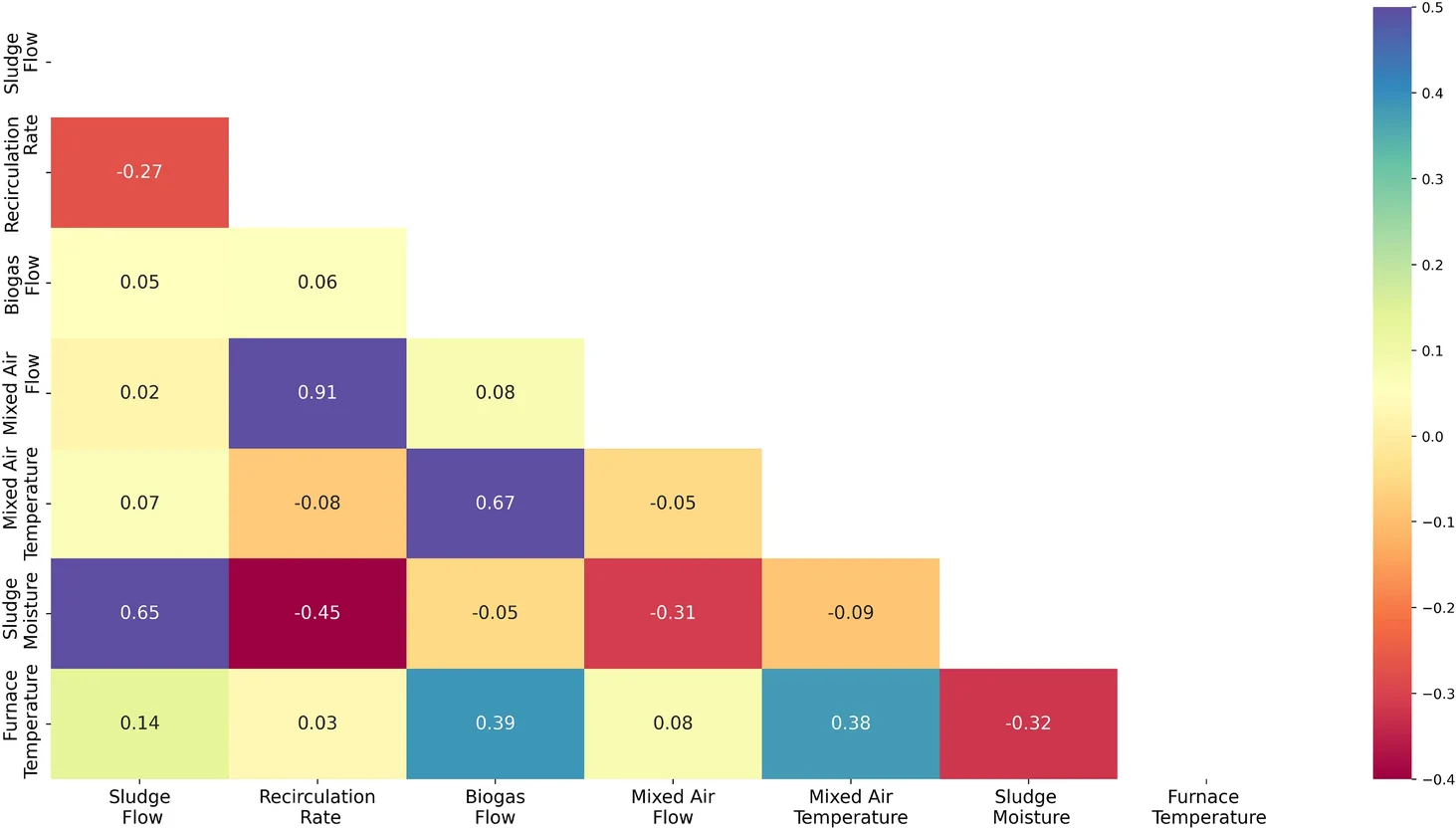
Pearson correlation.

The Pearson correlation analysis was conducted to investigate the relationships between various operational parameters, such as: sludge flow, recirculation rate, biogas flow, mixed air temperature, mixed air flow, moisture and furnace temperature. It should be noted that Pearson correlation captures only linear pairwise relationships and does not account for nonlinear interactions, causal dependencies, or potential confounding effects between variables. Therefore, the results should be interpreted as indication and possible trend and not a rigorous sensitivity or influence analysis. The correlation coefficients shown on the heatmap in Fig. 9 provide insight into the strength and direction of linear relationships between these parameters.

Starting from the negative correlations, the value of the Pearson correlation coefficient for the recirculation rate and the moisture of the sludge $\mathrm {(r=-0.45)}$ indicated that an increase in the recirculation rate is justified for processing the sludge with a lower moisture content. This correlation is evident owing to the fact of achieving higher combustion temperatures for less-moisturized fuels. A negative correlation is also observed between sludge moisture and furnace temperature $\mathrm {(r=-0.32)}$ and mixed air flow $\mathrm {(r=-0.31)}$. These values, suggesting a weak, although not yet negligible relation, show that lower moisture levels in the sludge contribute to higher process temperatures, and thus are associated with an increased need for combustion air to provide the required temperature level.

The positive correlation between recirculation rate and mixed air flow $\mathrm {(r=0.91)}$ of very high value shows strong linear dependence between these two. This obviously indicates that an increase in recirculation rate, related to its adjustments aimed to maintain optimal conditions within the system, corresponds with an increase in mixed air flow. A strong positive correlation is also shown between the biogas flow and mixed air temperature $\mathrm {(r=0.67)}$, suggesting that higher biogas flow rates translate into the elevated temperatures of the mixed air, owing to heat released during biogas combustion and the corresponding flue gas temperature rise. Another strong positive correlation coefficient was obtained for sludge feeding rate and sludge moisture $\mathrm {(r=0.65)}$, which clearly illustrates an increase of moisture in the system as the amount of sludge fed increases. Moreover, the moderate positive correlation between biogas flow and furnace temperature $\mathrm {(r=0.39)}$ suggests that as the biogas flow increases, the furnace temperature also tends to increase. Similarly to the mixed air temperature, this relationship can be explained by the fact that higher biogas flow can enhance combustion efficiency, and in consequence raise the furnace temperature. This interrelation is also expressed through the positive moderate value of the correlation coefficient between furnace temperature and mixed air temperature $\mathrm {(r=0.38)}$. Following this analysis, different scenarios in AnyLogic were considered based on the manipulation of the most influencing factors: biogas flow, sludge moisture, mixed air temperature and mixed air flow.

### Scenario analysis

In order to find the best solution for energy-saving in a wastewater treatment plant, the basic scenario, and five other proposed scenarios were simulated and compared. Each scenario characterised with different biogas flow rate, sludge moisture, mixed air temperature, and mixed air flow. These parameters were selected based on the Pearson correlation analysis described above. Based on the analysed options, the best proposed solution was selected.

The basic scenario is a simulation of the process with the same working conditions, as for the recorded data, used for both input data, and later the validation of the model. Further analysis includes 5 scenarios with parameters with parameters changed from the baseline (reference) scenario.

**Fig. 10:**
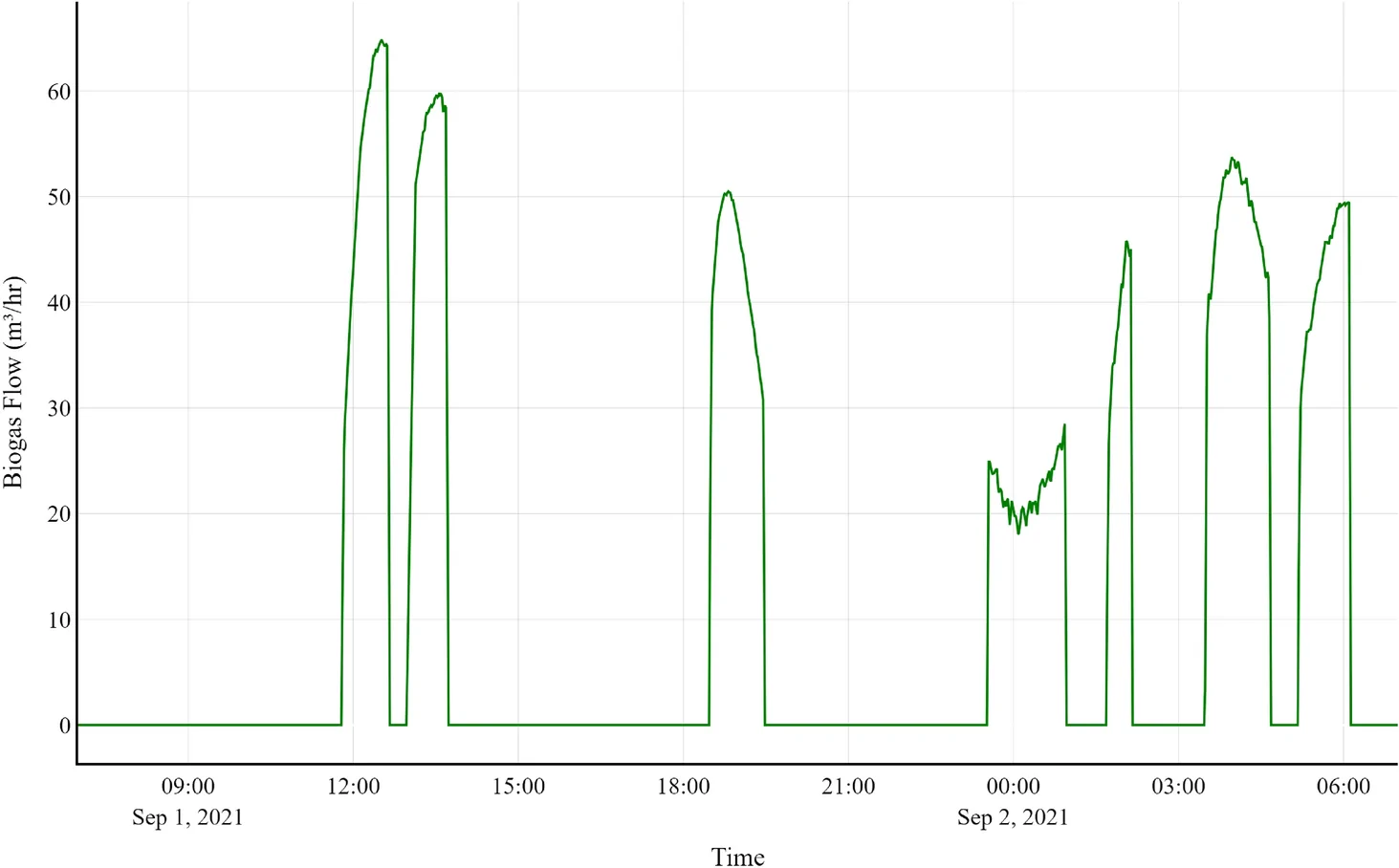
Daily fluctuations in biogas consumption $\mathrm {(m^3/h)}$ fed into the furnace.

The other proposed scenarios focus on reducing the costs and efficiency of the wastewater treatment plant with the optimisation of the biogas amount used in order to enhance the decomposition processes in the fluidized bed furnace. Since the reduction of the biogas flow rate causes the temperature drop in the furnace, the aim here was to find an optimal solution, where the biogas consumption is lowered, but the temperature in the furnace is kept above 850$^\circ$C, which is required for the correct and complete combustion, and satisfies the relevant regulations that involve the best available techniques (BAT) in the incineration of sewage sludge^41^. The daily fluctuations of biogas consumption in the real case scenario is presented in Fig. 10.

#### Multi-factor scenario

**Fig. 11:**
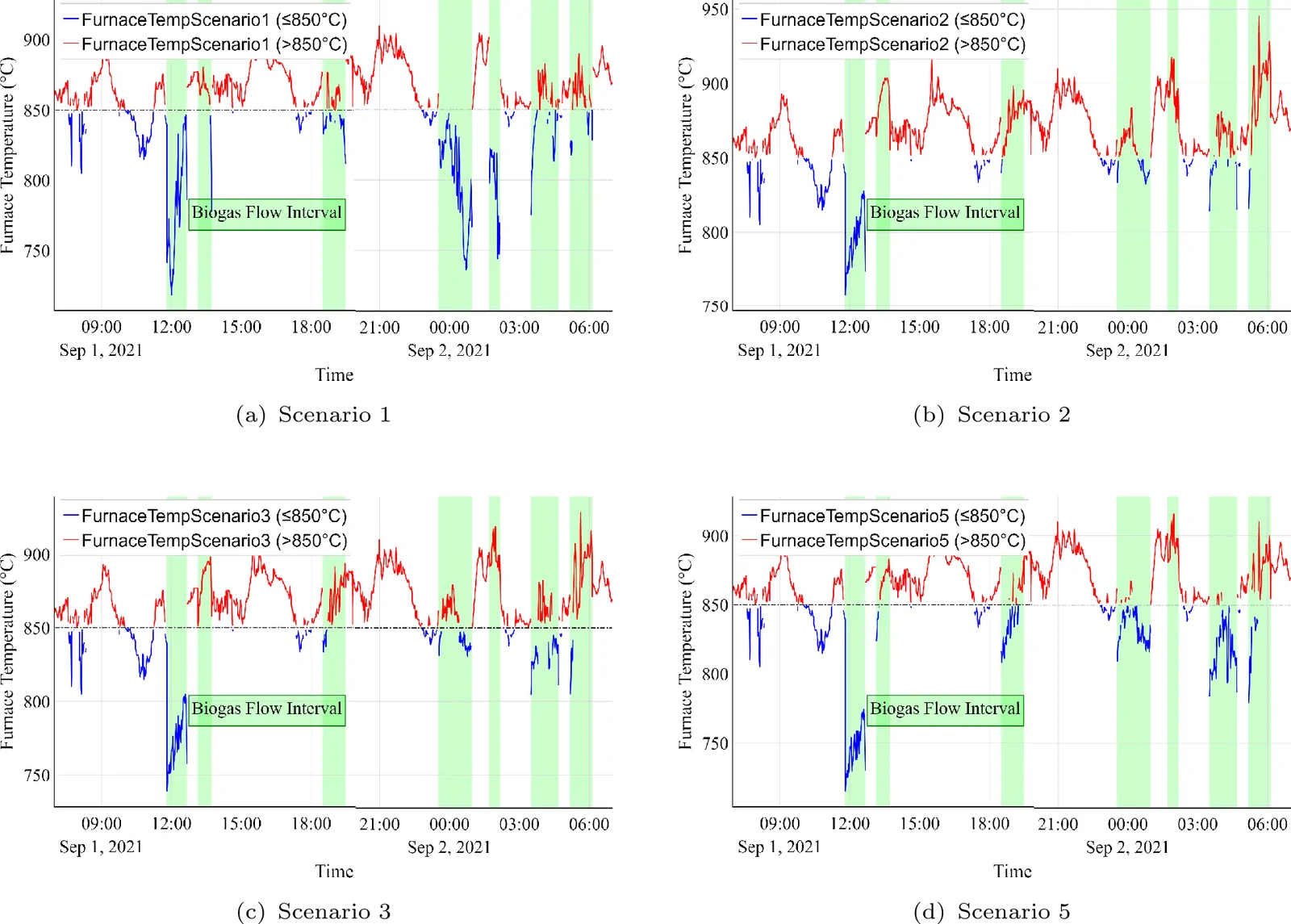
Furnace temperature variation for different scenarios.

**Fig. 12:**
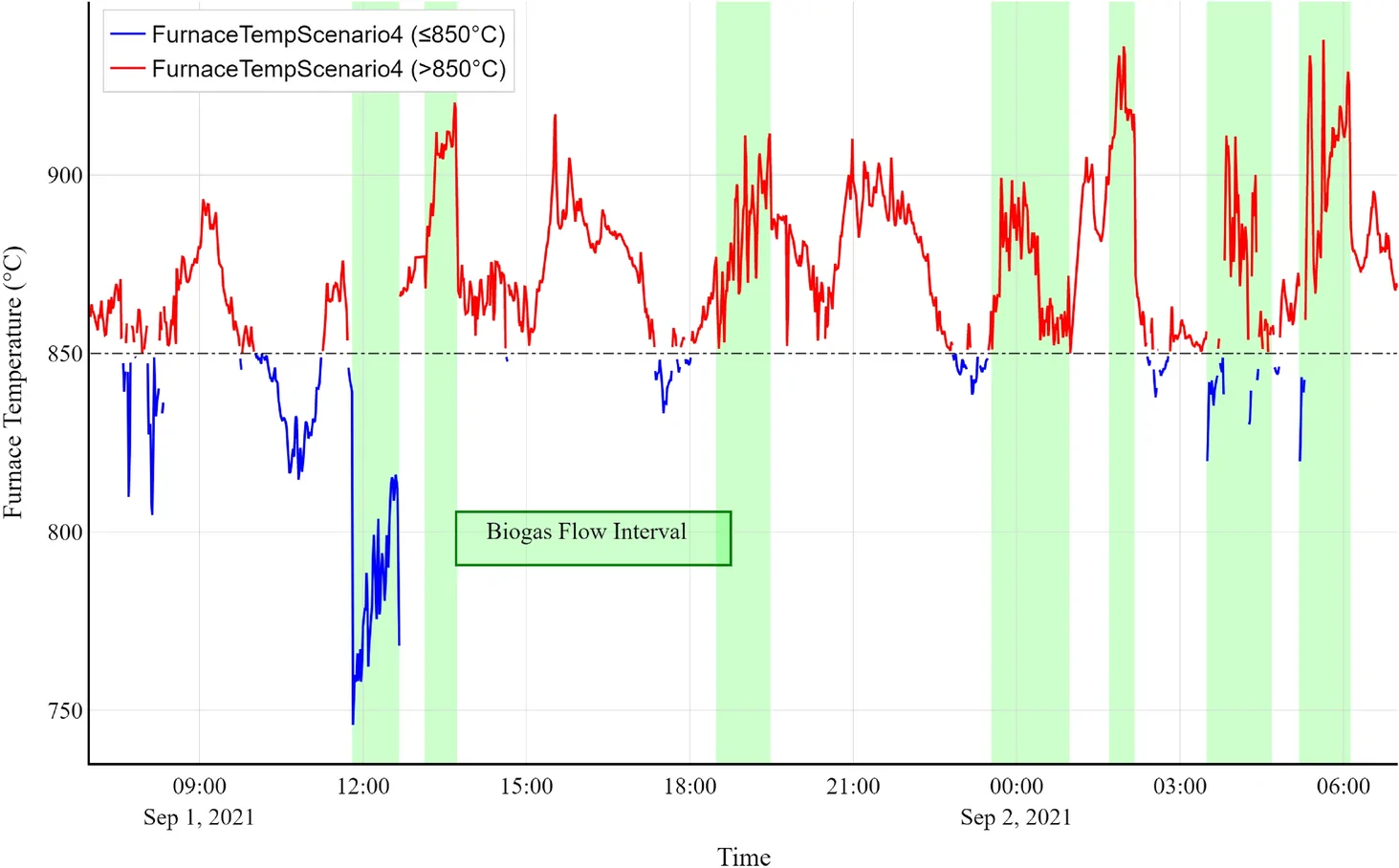
Furnace temperature variation for optimal scenario - scenario 4.

The change in the parameters for each proposed scenario is gathered in Table 1. The values are expressed in the percentage increase or decrease of the initial value of the parameter in the basic (reference) scenario, since the values varied in time in the 24 h period. The simulated temperature changes during this time are presented in Figures 11 and 12. The red lines represent temperatures over 850$^\circ$C, while the blue lines, below this value. Green areas indicate time periods, where the biogas was injected into the furnace in order to keep the required temperature level. The time in which the biogas was injected is the same for the basic scenario, and all of the other scenarios. The difference is the lower flow rate of the injected biogas, and therefore, lower total consumption.

**Table 1:** Scenario simulation plan (up and down arrows denote increase or decrease of the parameter).

Scheme	Biogas flow	Sludge moisture	Mixed air temperature	Mixed air flow
Scenario 1	30% *⇓*	5% *⇑*	20% *⇑*	10% *⇑*
Scenario 2	30% *⇓*	30% *⇓*	5% *⇓*	10% *⇑*
Scenario 3	40% *⇓*	35% *⇓*	10% *⇓*	15% *⇑*
Scenario 4	40% *⇓*	40% *⇓*	10% *⇓*	20% *⇑*
Scenario 5	45% *⇓*	40% *⇓*	20% *⇓*	15% *⇑*

The results across various scenarios show that in order to choose the favorable operating case involves a trade-off between the unacceptable operation duration and total biogas consumption. The unacceptable operation duration is the total time (within simulated 24 h), in which the temperature in the furnace dropped below 850$^\circ$C, which is the minimum combustion temperature demanded by European regulations. The basic scenario demonstrates 421 min of unacceptable operation duration and $\mathrm {273 m^3}$ of biogas consumption.

**Fig. 13:**
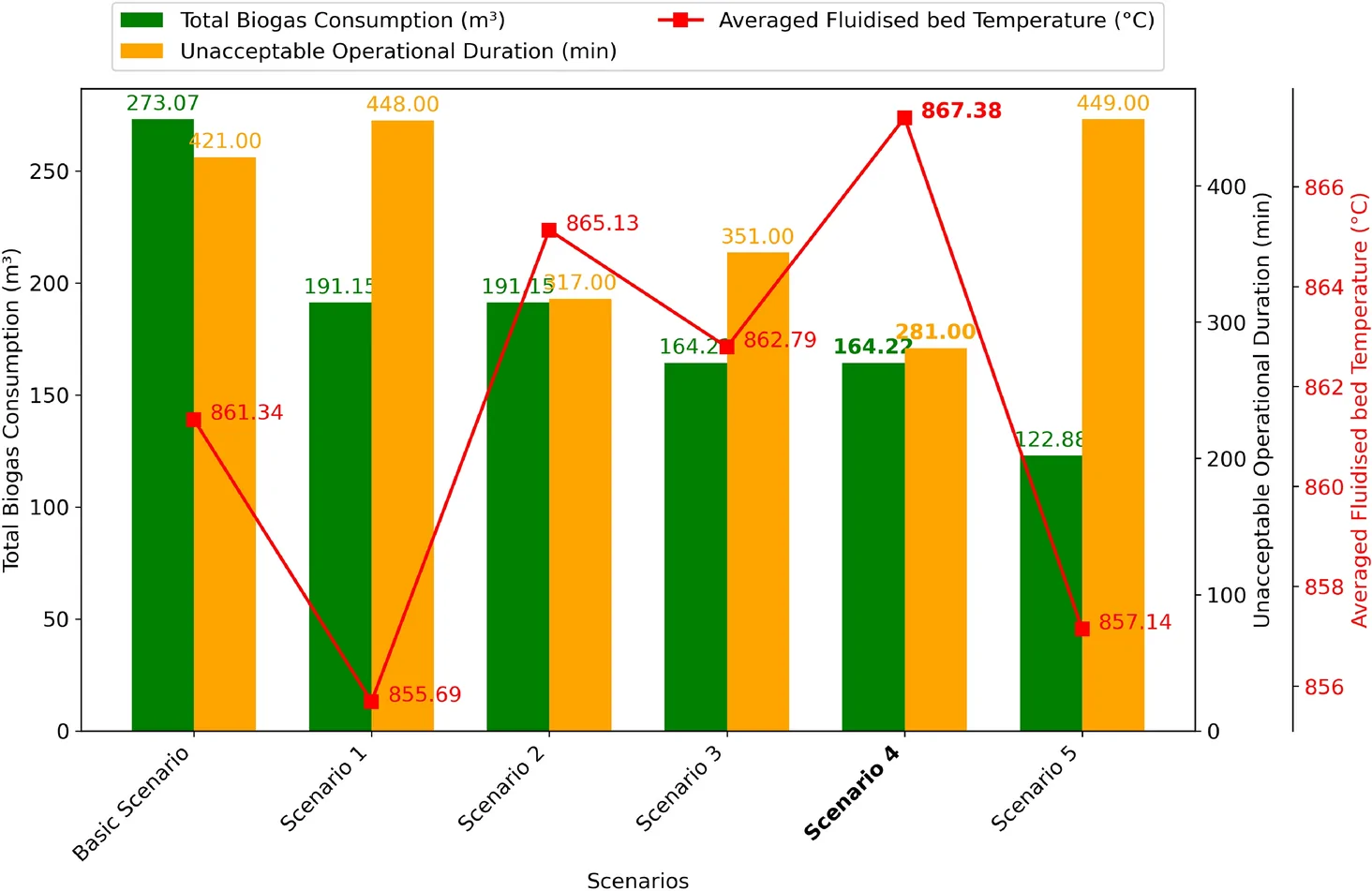
Comparison of operational metrics.

Scenario 1 assumed the decrease of 30% of biogas flow, and an increase in sludge moisture (5%), mixed air temperature (20%), and mixed air flow (10%), see Table 1. These changes resulted in a slightly increased unacceptable operation duration of 448 min, but with a significantly reduced biogas consumption of $\mathrm {191 m^3}$. For Scenario 2 it was also proposed to decrease the biogas flow by 30%, while the sludge moisture decreased by 30%, the mixed air temperature decreased by 5%, and the mixed air flow increased by 10%. This solution shows an improvement with a decreased unacceptable operation duration of 317 min, maintaining the biogas consumption at $\mathrm {191 m^3}$. Next, scenario 3, assumes a 40% decrease in biogas flow, a 35% decrease in sludge moisture, a 10% decrease in the mixed air temperature, and a 15% increase in mixed air flow. This continues the trend with further reduced unacceptable operation duration of 351 min, while biogas consumption lowered to $\mathrm {164 m^3}$. Scenario 4, by further decreasing the sludge moisture and inreasing the mixed air flow, presents an even more optimized performance, achieving 281 min of unacceptable operation duration and $\mathrm {164 m^3}$ of biogas consumption. Finally, scenario 5 attempts to further reduce biogas consumption (45% decrease). While showing the highest unacceptable operation duration of 449 min, scenario 5 achieves the lowest biogas consumption at $\mathrm {123 m^3}$. This showed that a further decrease in biogas flow causes the drop of the furnace temperature below the accepted level. Therefore, the best proposed scenario is scenario 4, which allows for both the reduction of the unacceptable operation duration and the biogas consumption. The biogas consumption was decreased by 40% when compared with the real-life case, and the unacceptable operation time decreased from 421 min to 281 min per day, which is 33% less. At the same time, the average fluidized bed temperature for scenario 4 is 867$^\circ$C, while for the basic scenario, it is 5 degrees lower (Fig. 13).

## Conclusions and final remarks

The study covers the analysis of a sewage sludge incineration system of the wastewater treatment plant and searches for possibilities of reducing costs and finding energy savings solutions for the existing real-life system. This work successfully demonstrated the development and implementation of a real-time digital twin for energy optimization in wastewater treatment, specifically targeting biogas consumption in the incineration process. By combining physical modeling, real-time data integration, and simulation in AnyLogic, we achieved a dynamic and accurate representation of the fluidized bed reactor and related components.

However, it should be noted that the model validation was performed using a single 24-h high-resolution dataset. Unfortunately, it is the only complete dataset available for this study. While this dataset captures daily variability, it does not reflect longer-term fluctuations such as seasonal effects, variations in sludge characteristics, or operational disturbances. Therefore, the presented results should be interpreted as a proof-of-concept demonstration rather than a fully validated industrial solution.

Five different scenarios with varying conditions were analysed using the developed model and the best solution predicted a 40% decrease in biogas consumption compared to real-life data (from 273 down to 164 $\mathrm {m^3}$). Simultaneously, the duration of low-temperature combustion processes was reduced from 421 to 281 min per day. It should be noted that, in the context of sludge incineration, maintaining temperatures above critical thresholds (e.g., 850 $^\circ$C) is essential. Therefore, model accuracy near this threshold is of particular importance and requires further investigation in future work. This solution indicates the potential for significant resource savings and improved efficiency of the WTTP’s energy use. However, to achieve this, the sludge moisture needs to be decreased by 40%. Since drying is an energy-consuming process, this must be considered to ensure the net benefits align with the plant’s energy optimization goals. This is an important direction for further research to support the development and extension of the presented study. In particular, a comprehensive energy balance of the system, including sludge dewatering, drying processes, and their energetic interaction with the incineration system, is required to properly assess the overall energy performance. Such an approach would allow evaluation of whether the reduction in biogas consumption corresponds to a net energy benefit or a shift in energy demand between process stages. However, the findings presented here underscore the potential of digital twin technology to significantly enhance energy efficiency, reduce operational costs, and contribute to more sustainable industrial processes.

The model presented in this work demonstrates the potential of a scenario-analysis framework for similar systems. It can be used as a great tool for analysing many possible solutions and alignments of the interrelating conditions and parameters of the system, reducing the need for expensive or time-consuming testing methods. The model can be easily adjusted and continuously updated with real-time data, while maintaining its accuracy and relevance over time. This adaptability is a key feature of digital twin technology, by making the model a base component for developing a digital twin of the system that enables dynamic monitoring, predictive analysis, and helps in decision making in a virtual environment mirroring real operations. Future work should focus on extended validation using longer and more diverse datasets, including different operating conditions, to assess model robustness. In particular, further analysis of residuals, potential bias, and model performance near critical operating thresholds will be necessary to support reliable real-time decision-making in industrial applications.

## Data Availability

The data that support the findings of this study are available from the corresponding author upon reasonable request.

## Appendix

**Table:**

Coefficient	Value		
$a_1$	67.399069	$f_1$	− 0.010030
$b_1$	− 0.286220	$f_2$	0.064924
$c_1$	2.714628	$f_3$	48.050224
$d_1$	3918.999760	$f_4$	51.002435
$e_1$	161.145341	$f_5$	0.000116
$a_2$	16.180075	$f_6$	− 0.320674
$b_2$	0.000011		
$c_2$	− 0.009688		
$d_2$	3528.291102		
$e_2$	− 690.424030		
$f_2$	0.064924		
$f_3$	48.050224		
$f_4$	51.002435		
$f_5$	0.000116		
$f_6$	− 0.320674		
$f_7$	− 0.040971		
$f_8$	− 2.526874		
$f_9$	0.247516		
$f_{10}$	240.928215		

## List of symbols

$\dot{m}$: Mass flow rate, kg/s
*T*: Temperature, K
$$\dot{Q}$$: Energy flux, W
*λ*: Excess air ratio

## Subscripts and superscripts

$$\textrm{a}$$: Air
$$\textrm{amb}$$: Ambient, fresh
$$\textrm{ch}$$: Chemical
$$\textrm{d}$$: Dry
$$\textrm{fg}$$: Flue gas
$$\textrm{frn}$$: Furnace
$$\textrm{HEX}$$: Heat exchanger
*in*: Inlet
$$\textrm{mix}$$: Mixed
*out*: Outlet
$$\textrm{ox}$$: Oxidizer
*r*: Recirculated
$$\textrm{st}$$: Steam
$$\textrm{ss}$$: Sewage sludge

## Acronyms

$$\textrm{DT}$$: Digital twin
$$\textrm{HEX}$$: Heat exchanger
$\textrm{WHR}$: Waste heat recovery
$\textrm{WWTP}$: Wastewater treatment plant
